# Pulmonary alveolar microlithiasis

**DOI:** 10.4103/0970-2113.80334

**Published:** 2011

**Authors:** H. J. Gayathri Devi, K. N. Mohan Rao, K. M. Prathima, Jayanth K. Das

**Affiliations:** *Department of Chest Diseases, MS Ramaiah Medical College, Bangalore, India*; 1 *Department fo Pathology, MS Ramaiah Memorial Hospital, Bangalore, India*; 2 *Department of Cardiothoracic Surgery, MS Ramaiah Narayana Hrudayalaya, Bangalore, India*

**Keywords:** Pulmonary alveolar microlithiasis, misdiagnosis, miliary tuberculosis, biopsy

## Abstract

Pulmonary alveolar microlithiasis is a rare disease of unknown cause. We report a case in a young boy who presented with history of failure to thrive and chest X-ray finding suggestive of miliary mottling. Open lung biopsy revealed pulmonary alveolar microlithiasis.

## INTRODUCTION

Pulmonary alveolar microlithiasis (PAM) is an uncommon idiopathic disease characterized by microliths in the alveoli, and was first described by Friedrich in 1856 and then by Harbitz in 1918.[1,2] In 1957, Sosman emphasized that 50% of the cases were familial.[3] It is regarded as an autosomal recessive lung disease. As of 2004, 576 PAM cases have been studied, and most have originated from Europe (42.7%) and Asia (40.6%). Pulmonary tuberculosis or sarcoidosis was misdiagnosed in 88 cases of the 576.[4]

In this report, our aim is to emphasize a high index of suspicion for this uncommon disorder to avoid misdiagnosis of miliary tuberculosis.

## CASE REPORT

A fifteen-year-old boy was investigated for failure to thrive. He was totally asymptomatic and no respiratory complaint at the time of evaluation. He had received two courses of anti tuberculosis treatment (2007 and 2009) elsewhere with the diagnosis of Miliary tuberculosis based on radiological findings. Clinical examination did not reveal any abnormality.

### Investigations

**Figure 1:**
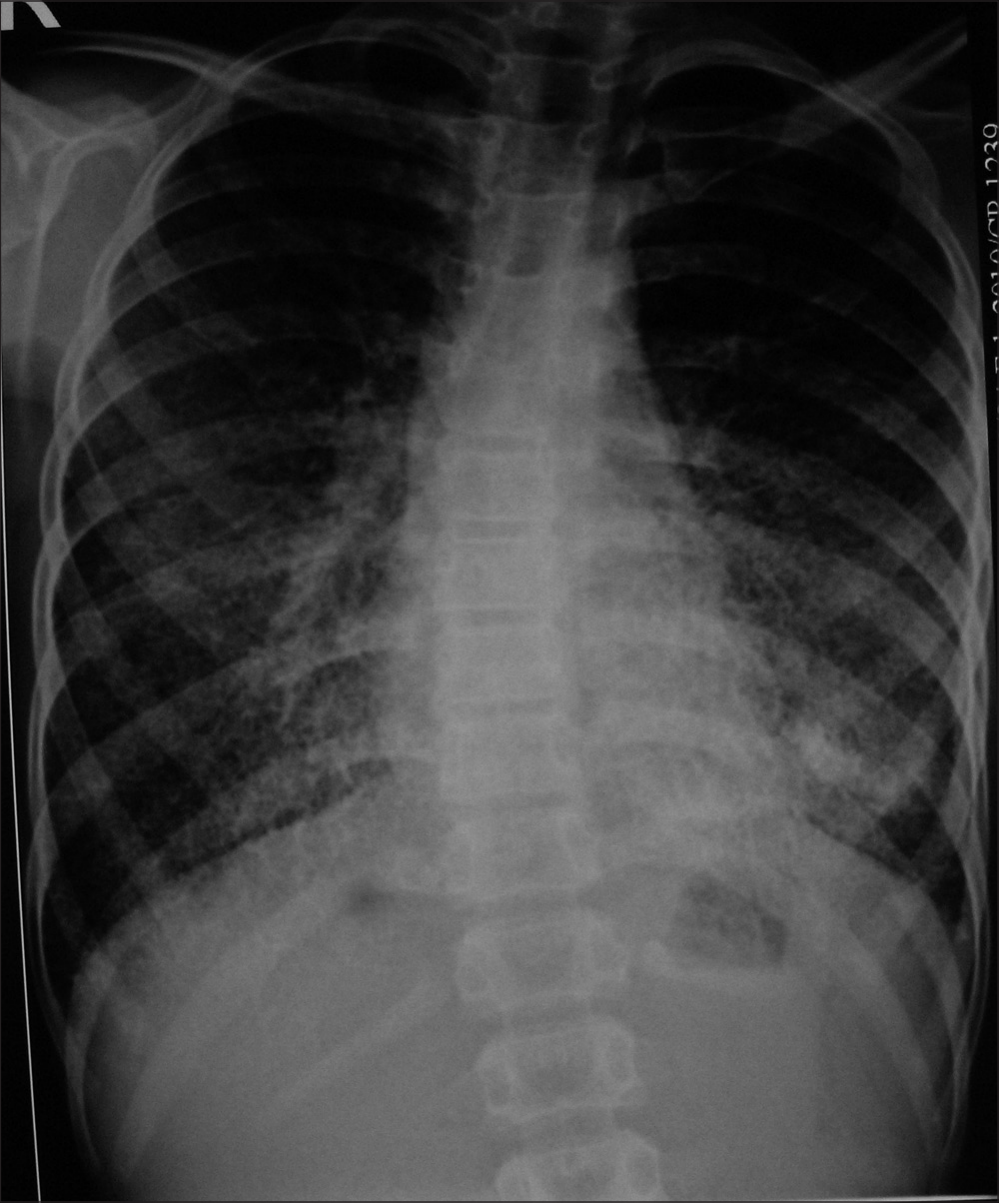
Chest X-ray showing bilateral diffuse high density micronodular opacities more toward the mid and lower zones

**Figure 2:**
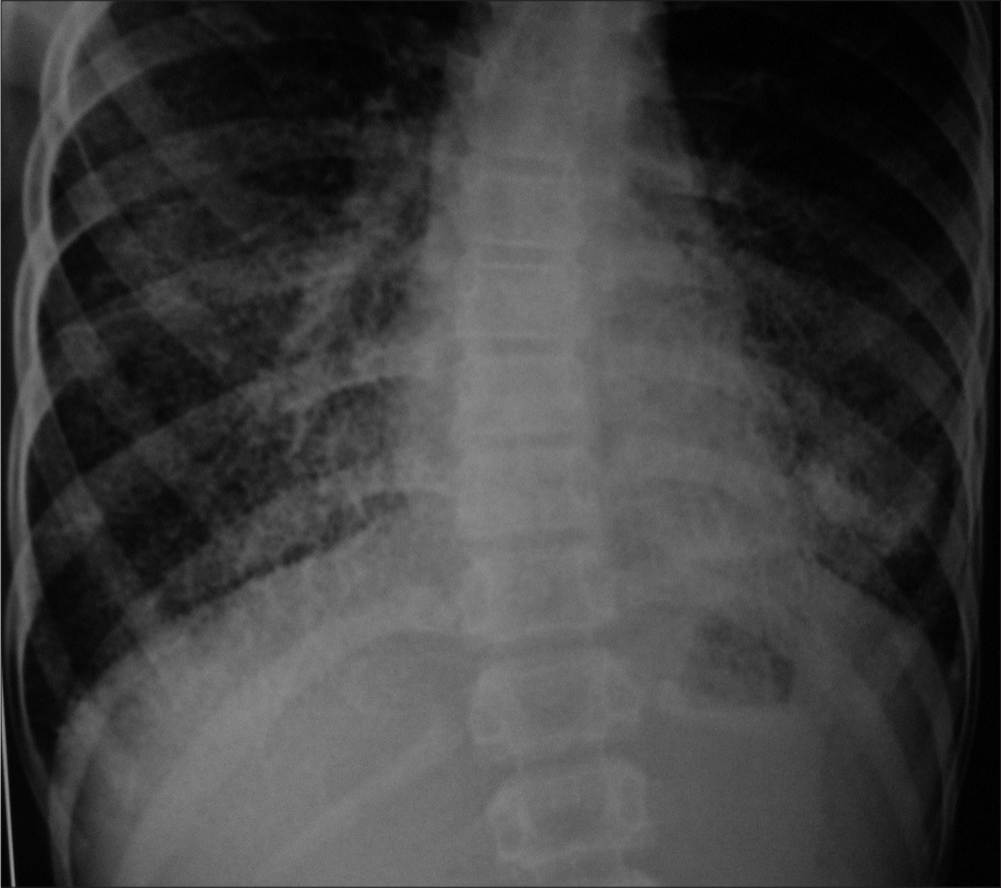
Chest X-ray showing bilateral diffuse high density micronodular opacities more toward the mid and lower zones

**Figure 3:**
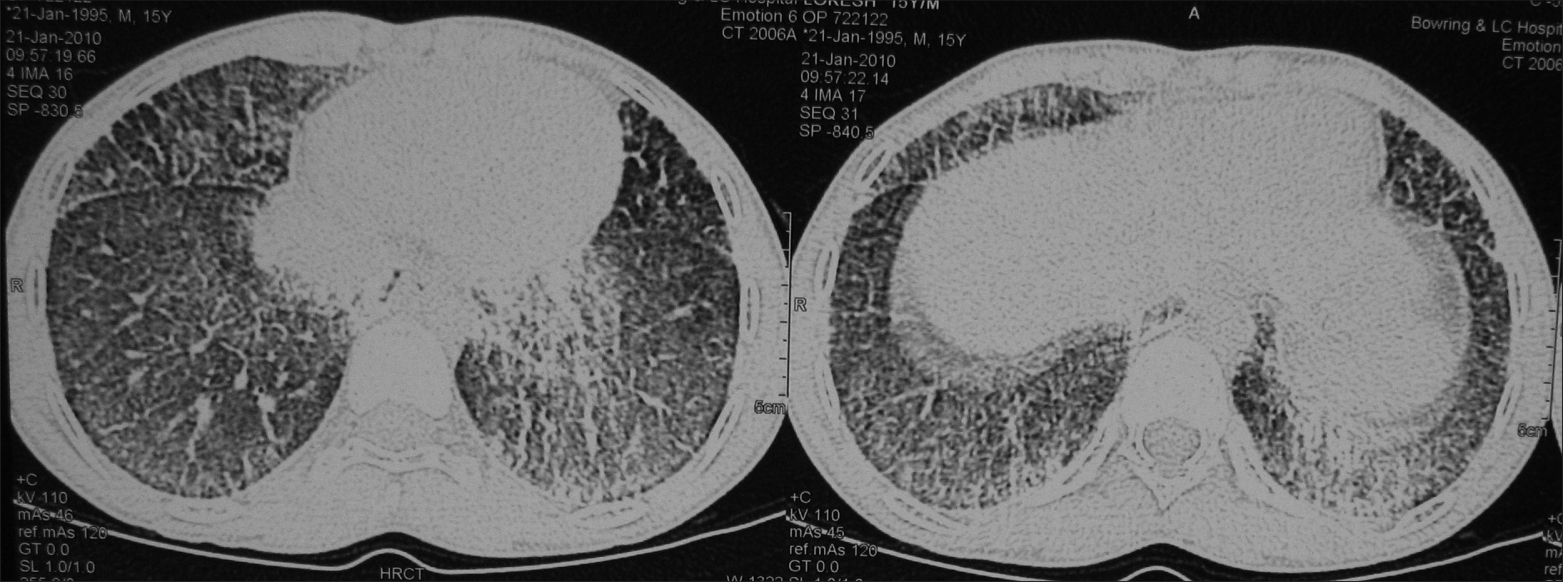
CT of Thorax showing bilateral diffuse micronodular opacities distributed predominanly over basal and posterior regions with reticular pattern

**Figure 4:**
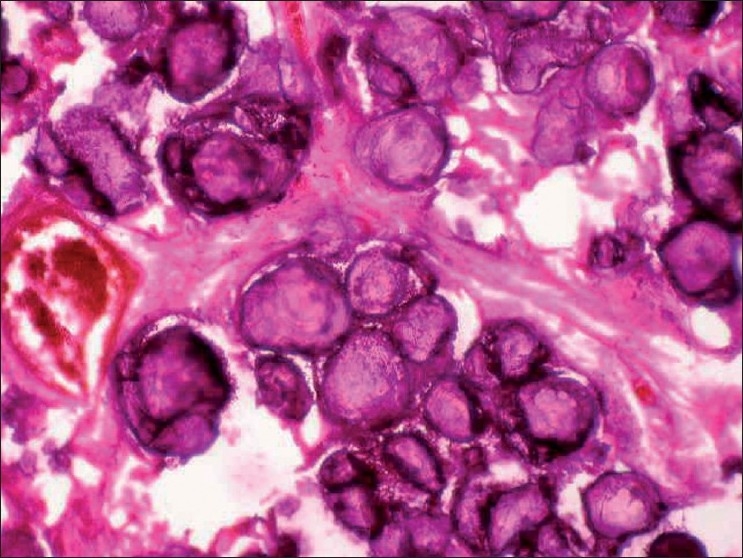
Histopathology section showing multiple calcispherites within the alveoli of lung parenchyma. H and E 100×

His CBC, electrolytes and calcium were normal. Ultrasound of abdomen was normal. Flow volume loop showed mild restriction. Serial chest X-rays from 2007 showed bilateral diffuse micronodular calcific shadows more toward the mid and lower zones [Figures 1 and 2]. HRCT scan showed bilateral diffuse micronodular opacities distributed predominantly over basal and posterior regions with thickening of interlobar septa [Figure 3]. Wedge biopsy of the Lingula was done. Histopathology examination revealed multiple calcispherites within the alveoli of lung parenchyma suggestive of pulmonary alveolar microlithiasis of lung [Figure 4]. Chest X-ray of his only sibling did not reveal any abnormality.

## DISCUSSION

PAM is an uncommon disease characterized by widespread localization of calcispherites (salts of calcium and phosphate) in the alveolar spaces in the absence of any known disorder of calcium metabolism. Alveolar microlithiasis is reported worldwide and one-quarter of patients are from Turkey.[5] PAM can be seen in any age group. Sex distribution is equal. A familial incidence particularly in siblings is common. Case reports of PAM have been published in Indian literature.[6]

PAM has been described in detail by Sosman in 1957. Recently, the gene SLC34A2 has been identified and is considered to be responsible for PAM. This gene codes for type IIb sodium-dependent phosphate transporter and its function may provide insight into the pathogenesis of this disorder. Confirmation of diagnosis of PAM can be done by demonstrating the mutation in the SLC34A2 gene.[7]

A striking feature of this disease is lack of significant symptoms despite extensive radiographic changes. Patients may remain asymptomatic for many years and usually become symptomatic between third and fourth decades, the condition may progress slowly leading to progressive dyspnoea with or without cough and ultimately end up with respiratory insufficiency and cor pulmonale. Plain chest radiographs usually reveal diffuse bilateral areas of micronodular calcifications that predominate in the middle and lower lung areas.

There is no known medical treatment to reduce or halt the progression of the disease. Palliative treatments with systemic corticosteroids, calcium-chelating agents and serial bronchopulmonary lavage have been shown to be ineffective. Attempts to reduce calcium phosphate precipitation in pulmonary alveoli has been tried with diphosphonate[8] Lung transplantation remains the only possible treatment for end-stage cases.[9]
